# First report on a *Raillietiella orientalis* and *Kalicephalus* sp. co-infection in a wild-caught red-tailed green rat snake (*Gonyosomaoxycephalum*)

**DOI:** 10.1016/j.ijppaw.2026.101240

**Published:** 2026-05-23

**Authors:** Paula Sapion-Miranda, Ulrich Gärtner, Anja Taubert, Carlos Hermosilla, Fanny Kratz, Malek J. Hallinger

**Affiliations:** a Institute of Parasitology, Justus Liebig University Giessen, Giessen, Germany; b exomed GmbH, Marburg, Germany; c Royal Museum for Central Africa (BopCo), Tervuren, Belgium; d Institute of Anatomy and Cell Biology, Justus Liebig University Giessen, Giessen, Germany

**Keywords:** Reptiles, Parasites, Exotic pets, Reptile medicine, Wildlife, Pentastomids, Emerging diseases

## Abstract

Parasitic diseases are a major but frequently overlooked health concern in reptiles, with consequences that extend beyond individual health issues to broader ecological and conservation concerns. A male red-tailed green rat snake (*Gonyosoma oxycephalum*), imported from Indonesia, died in quarantine one month after purchase, lacking any signs of illness except for a brief period of anorexia. Following its death, the owner observed a vermicular parasite emerging from the snake's oral cavity, which prompted submission of the animal to necropsy. A high-grade infection with 40 specimens of the pentastomid *Raillietiella orientalis* was confirmed by morphological and DNA analyses (18s rRNA). Moreover, histological examinations revealed macroscopic and histological evidence of larval migration. This case represents the first documented infection in red-tailed green rat snakes and signifies one of few reports on *R. orientalis* for Europe. It illustrates the potential introduction of invasive parasites through the exotic pet trade in the absence of adequate pre-import health screening. While the risk of establishment in European ecosystems remains uncertain, such introductions may pose challenges for captive reptiles, zoological collections and the native fauna. In addition, co-infections may alter disease progression and contribute to the pathogenicity of pentastomid infections.

## Highlights

• The third confirmed imported European case of *Raillietiella orientalis*.
• Severe pathogenic effects of high-intensity *R. orientalis* infection.
• The pet trade remains an under-monitored pathway for emerging infectious diseases.

## Graphical abstract

**Figure:**
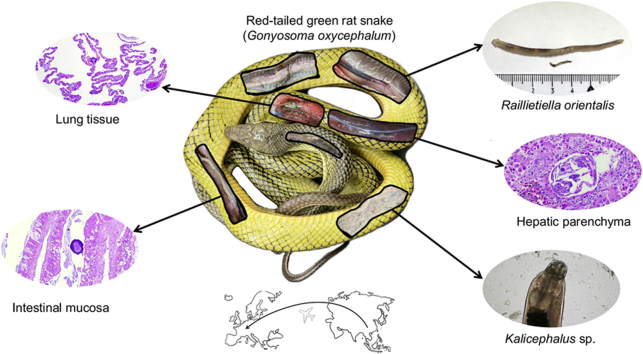

## 1 Introduction

Pentastomids represent a phylogenetically basal lineage of crustacean-related endoparasites which primarily infect reptiles as definitive hosts (Riley, 1986; Paré, 2008; Christoffersen and De Assis, 2013; Kelehear et al., 2014). Despite an extensive fossil record dating back to the Late Cambrian (∼500 million years ago), they remain relatively understudied in the context of reptile pathology (Walossek and Müller, 1994; Maas and Waloszek, 2001; Paré, 2008; Kelehear et al., 2014; Swanepoel et al., 2022). These metazoans follow a heteroxenous life cycle, involving a variety of intermediate hosts like mammals, rodents, lizards, anurans, fish, and insects (Riley, 1986; Paré, 2008; Kelehear et al., 2014; Farrell et al., 2025). Invasive pentastomiasis is among emerging infectious diseases diminishing native reptile populations worldwide (Olson et al., 2024; Palmisano et al., 2025a). In this context, *Raillietiella orientalis* (Hett, 1915) is recognized for its euryxenous host range and has been reported to spread across continents (Kelehear et al., 2014; Miller et al., 2018; Walden et al., 2020; Farrell et al., 2023; Palmisano et al., 2025a). In Florida, a well-documented hotspot for invasive species and emerging infectious diseases due to its favorable climate (Searcy et al., 2023), the parasite was initially detected in Burmese pythons (*Python bivittatus*) introduced via pet trade (Miller et al., 2018) and has since then become established in over 18 native snake species (Miller et al., 2020; Palmisano et al., 2025a,Palmisano et al., 2025b).

**Fig. 1:**
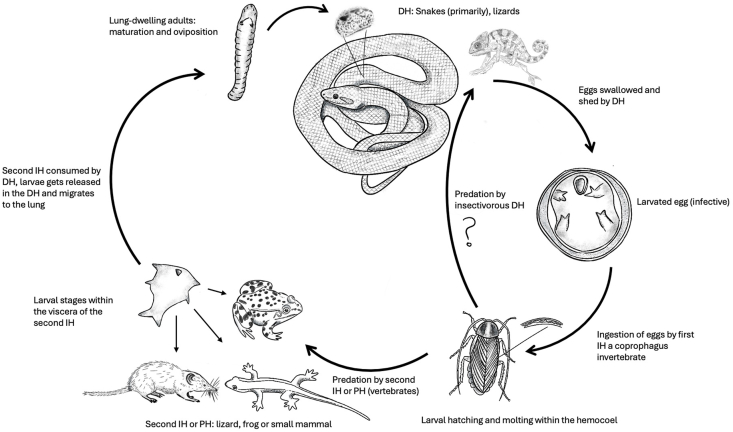
Presumed life cycle of *Raillietiella orientalis*. DH = Definitive host; IH= Intermediate host; PH= Paratenic host (according to Lavoipierre and Rajamanickam, 1973; Ali and Riley, 1983; Bosch, 1986; Palmisano et al., 2022).

Specifically, *R. orientalis*, like other raillietiellid species, uses cockroaches (Blattodea) as first intermediate host (e.g., *Blaberus discoidalis*) (Lavoipierre and Rajamanickam, 1973; Ali and Riley, 1983; Bosch, 1986; Palmisano et al., 2022). Experimental and field observations further suggest a complex multi-host lifecycle involving amphibians and reptiles as secondary intermediate or paratenic hosts prior to infecting the definitive host (Miller et al., 2020; Palmisano et al., 2022; Farrell et al., 2025). Considering the predominantly vertebrate-based diet of snakes, anurans, reptiles and small mammals are presumed to play a particularly important role in transmission dynamics (Miller et al., 2018; Palmisano et al., 2022; Farrell et al., 2025). In recent years, *R. orientalis* has also been documented captivity-kept insectivorous reptiles acting as definitive hosts in Europe, including a Meller's chameleon (*Triocerus melleri*) in Germany (Sapion-Miranda et al., 2025) and a panther chameleon (*Furcifer pardalis*) in Belgium (Hellebuyck et al., 2025). These cases raise questions regarding the parasite's life cycle and the possibility of its establishment outside its currently recognized range. The presumed complex multi-host life cycle is illustrated in Fig. 1.

Due to its pronounced phenotypic plasticity, this parasite can infect a broad spectrum of hosts, facilitating adaptation to novel environments and continuous range expansion (Miller, 2017; Westfall et al., 2019). While the zoonotic potential of *R. orientalis* remains unknown (Farrell et al., 2023), several pentastome genera have been confirmed as zoonotic, including *Armillifer*, *Porocephalus* and *Raillietiella* (Tappe and Büttner, 2009; Pantchev and Tappe, 2011; Tappe et al., 2016; Mendoza-Roldan et al., 2022).

The red-tailed green rat snake (*Gonyosoma oxycephalum*) is a colubrid species native to Southeast Asia, where it occupies arboreal niches and preys upon on bats, rodents and birds (Dieckmann et al., 2015; Dela Cruz et al., 2018). Despite its prevalence, relatively little is known regarding its endoparasite fauna and existing reports are largely limited to other Southeast Asian snakes within the family Columbridae (Chaiyabutr and Chanhome, 2002; Yudhana et al., 2023). Due to its striking appearance, *G. oxycephalum* is a popular species in the exotic pet trade with a significant number of individuals sourced directly from wild populations (Dieckmann et al., 2015; Kar Men et al., 2022).

This report presents the first documented case of a severe co-infection of *R. orientalis* and *Kalicephalus* sp. in a wild-caught red-tailed green rat snake (*Gonyosoma oxycephalum*), a host species not previously reported for *R. orientalis* infections, and the third case documented so far in Europe (Sapion-Miranda et al., 2025; Hellebuyck et al., 2025). Besides documenting host novelty, this case report provides new insights into the systemic pathology of infection, as demonstrated by histological analysis. These findings reveal that, due to somatic parasite migration, *R. orientalis* causes extensive tissue damage affecting multiple organs, thereby highlighting the parasite's pathogenic potential (Paré, 2008; Miller et al., 2018, 2020; Hellebuyck et al., 2025; Graham et al., 2026). Furthermore, this case underscores the importance of quarantine periods, regular surveillance, and early detection of neglected parasitic infections, which may have implications for both reptile and human health (Murray et al., 2025). Given that the endoparasite fauna of *G. oxycephalum* is currently almost unknown, providing detailed data on this subject is essential for understanding host-parasite dynamics. By detailing organ effects and the consequences of co-infection, this report contributes valuable knowledge for future research on host–parasite interactions, pathogenesis, and the potential ecological risks associated with invasive parasite introduction via the wildlife trade.

## 2 Material and methods

### 2.1 Case description

A male adult red-tailed rat snake (*G. oxycephalum*), which was originally captured in Borneo, Indonesia, was purchased by a private owner in Germany. The owner had reportedly bought the exotic snake from a Czech reptile trader. After being purchased abroad, the animal was housed in quarantine after its arrival in Germany in October 2024. One month later, the animal suddenly died without exhibiting apparent clinical signs, besides a brief period of anorexia that was noted by the owner but not assessed by a veterinarian. After the animal's death, the owner noticed a ‘worm-like’ parasite egressing from the snake's mouth. This specimen was collected along with the snake and rapidly submitted to exomed GmbH to determine both the parasite species and cause of death.

### 2.2 Pathological examination, histology and microbiology

The snake was necropsied based on previously described procedures (Hallinger et al., 2020; Stacy, 2021). Several tissue samples (trachea, lung, liver, intestine and kidneys) were fixed in 10% neutral buffered formalin, embedded in paraffin, stained with haematoxylin and eosin (H&E), and studied using a light microscope (Axiolab 5, Carl Zeiss Microscopy GmbH, Germany). Fecal samples were also taken *post-mortem* and examined by both direct saline fecal smears (DSFS) and flotation-sedimentation method with Sheather's sugar solution (specific gravity = 1.27), which was prepared in-house following standard protocols (Zajac and Conboy, 2021). Moreover, swabs from the liver were collected for microbiological analysis, including bacterial and fungal culture. Columbia sheep blood agar (5%), MacConkey agar, and Sabouraud dextrose agar (SDA) were used for *in vitro* cultivation (all Oxoid Deutschland GmbH, Germany). Bacterial isolates were further characterized by Gram staining, oxidase and catalase testing (MAST Diagnostica GmbH, Germany), and identified by commercially available API 20 E/NE kits (BioMerieux, Charbonnier les Bains, France), as previously described in reptiles (Marenzoni et al., 2015; Hallinger et al., 2019).

### 2.3 Pentastomid identification, DNA extraction and amplification

Two adult pentastomid specimens were preserved in 70% ethanol for subsequent DNA-based species identification, which analysis aimed at validating the morphological identification performed using identification key accordingly (Brookins et al., 2009).

A tissue sample was taken from the largest ethanol-preserved specimen by excising a piece of the external body surface, avoiding opening of the digestive tract to prevent contamination with host and other alien DNA. Genomic DNA was extracted using the DNeasy Blood & Tissue Kit (Qiagen, the Netherlands), following the manufacturer's instructions and with a final elution volume of 60 μL. Polymerase chain reaction (PCR) amplification targeted the small subunit ribosomal RNA gene (18S), using the pentastomid-specific primer pair Pent629F–Pent1011R (Brookins et al., 2009). The PCR reaction was carried out in a total volume of 18 μL, containing 2.5 μL of DNA template, 1.8 μL of 10X buffer, 0.4 μM of each primer, 1.5 mM of MgCl_2_, 0.2 mM of dNTPs, and 0.03 units/μL of Platinum™ Taq DNA Polymerase (Invitrogen™, USA). A negative control was included using the same PCR master mix composition, but with ultrapure water added instead of template DNA.

Thermocycling conditions followed the protocol optimized by Brookins et al. (2009), with an initial denaturation step at 94 °C for 5 min, followed by 40 cycles of 94 °C for 30 s, 58 °C for 1 min, and 72 °C for 1 min, and a final extension step at 72 °C for 7 min. PCR products and negative controls were checked on a 1.3% agarose gel using a UV transilluminator and the MidoriGreen™ Direct (NIPPON Genetics, Germany) method.

Amplicons were subsequently purified using the ExoSAP-IT™ protocol (following manufacturer's instructions) and bidirectionally sequenced using the BigDye® chemistry (Macrogen™, Amsterdam). The resulting sequences were trimmed to remove the primers and assembled into consensus sequences using Geneious Prime(R) (v2019.2.3; Biomatters Ltd., New Zealand). The consensus sequence was then compared to the GenBank DNA reference database using the Basic Local Alignment Search Tool (BLAST) (Altschul et al., 1990). Additionally, a Neighbor-Joining tree was constructed using the Tamura–Nei substitution model with 1000 bootstrap replicates. To this end, all available 18S sequences at the Pentastomida level were downloaded from GenBank, cleaned, and aligned with our generated sequence and one outgroup [Accession number: DQ639844; *Acanthogonatus nahuelbuta* (Araneae)] using MUSCLE in Geneious Prime. The final alignment included 42 18S sequences and was trimmed to retain a 404 bp overlapping region.

### 2.4 Scanning electron microscopy (SEM) analysis

SEM imaging was performed on one adult female specimen and one adult male specimen of *R. orientalis*, as well as on one female *Kalicephalus* sp. All specimens were initially fixed in 2.5 % glutaraldehyde and post-fixed with 1 % osmium tetroxide (both Merck, Darmstadt, Germany). After rinsing in distilled water, the samples were dehydrated in a graded ethanol series, subjected to critical point drying using CO_2_, and finally sputter-coated with gold. SEM analysis was performed using a Philips XL30 scanning electron microscope.

## 3 Results

**Fig. 2:**
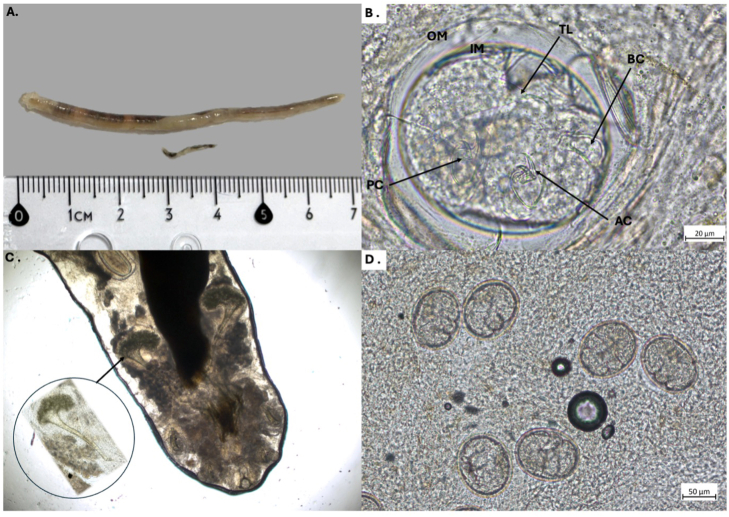
Morphology of *Raillietiella orientalis***A.** Sexual dimorphism in *R. orientalis*, the female is markedly larger than the male **B.** Fully developed larva within the egg, showing the eggs outer membrane (OM) and inner membrane (IM). In the egg, the larval buccal cadre (BC) is visible, as well as the limb region bearing anterior claws (AC) and posterior claws (PC) with sclerotized hooks, terminating in the tail (TL). **C.** Male *R. orientalis* showed characteristic prominent spicules, marked by a raised reticulum (circled magnification and arrow). **D.***R. orientalis* eggs observed in native fecal smears from rectum content.

**Fig. 3:**
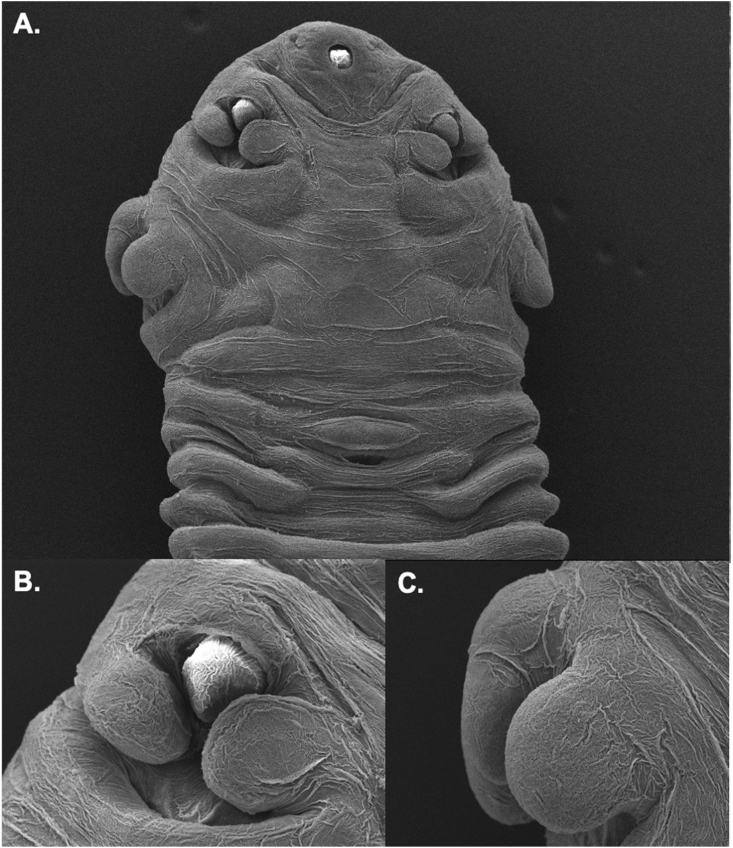
Scanning electron microscopy (SEM) illustration of the cephalothorax of *Raillietiella orientalis*, collected from a red-tailed green snake (*Gonyosoma oxycephalum*). **A.** Ventral view of the cephalothorax showing the oral (white arrow) and anal openings (red arrow), as well as the four hook pits. **B.** Close-up of an anterior hook (AH) with adjacent cephalic papillae (CP). **C.** Close-up of a posterior hook, which appears retracted and covered by cephalic papillae.

Overall, 40 adult pentastomids were found within the coelomic cavity of the red-tailed green rat snake during necropsy, of which 19 were male and 21 female specimens. The typical sexual dimorphism was evident since males measured 8 to 11 mm (mean 9.5 mm), whilst females ranged from 33 to 65 mm (mean 49 mm) (Fig. 2A). Referring to parasite egg diagnosis, the embryonated eggs detected in faecal samples of the snake exhibited morphological characteristics of the order Cephalobenidae, as the larvae showed deep buccal furca (Fig. 2B), thereby distinguishing them from members of the order Porocephalida (Frank, 1986; Do Nascimento et al., 2020). The embryonated eggs containing larvae exhibited active movement (Additional file 1: supplementary video). These eggs measured 97.57 μm in length (Fig. 2B). Moreover, the adult pentastomids were morphologically consistent with the genus *Raillietiella* (Ali and Riley, 1983; Bosch, 1986). Hence, microscopic examination of the male specimen revealed the characteristic elevated reticulum and tubular spicules, which are typical for *R. orientalis* (Ali et al., 1985) (Fig. 2C). Parasitological examination of native fecal smears revealed a high parasite burden, with approximately 30 eggs per slide (Fig. 2D). In contrast, the sedimentation–flotation method using Sheather's sugar solution detected only 3–5 eggs per slide. Notably, the SEM images presented here provide the first documentation of the surface fine structure of a male *R. orientalis* (Fig. 3). Furthermore, the comparative genetic analyses based on 18s rRNA sequences revealed 100% sequence identity between current parasites and *R. orientalis* thereby confirming morphology-based findings.

Supplementary video related to this article can be found at https://doi.org/10.1016/j.ijppaw.2026.101240

The following is/are the supplementary data related to this article.

Multimedia component 1

During snake necropsy, the adult pentastomids were found distributed throughout the total coelomic cavity. Active movement of the parasite within the host was observed (Additional file 2: supplementary video). Hence, a total of six females were located within the tracheal lumen, with additional three females positioned peritracheally. One *R. orientalis* specimen was found in the pericardial region. Within the lung tissue, four males and two females were identified. Along the lateral coelomic wall, cranial to the liver, a single specimen was present. From the serosal surface of the liver, caudal to the kidneys, two females and four males were observed. Between the kidneys and the cloaca, three females and four males were detected. In the rectal region, seven males were present. All pentastomids were attached to the serosal surfaces of organs throughout the coelomic cavity, except for those embedded within the lung tissue and those located intratracheally.

Supplementary video related to this article can be found at https://doi.org/10.1016/j.ijppaw.2026.101240

The following is/are the supplementary data related to this article.

Multimedia component 2

**Fig. 4:**
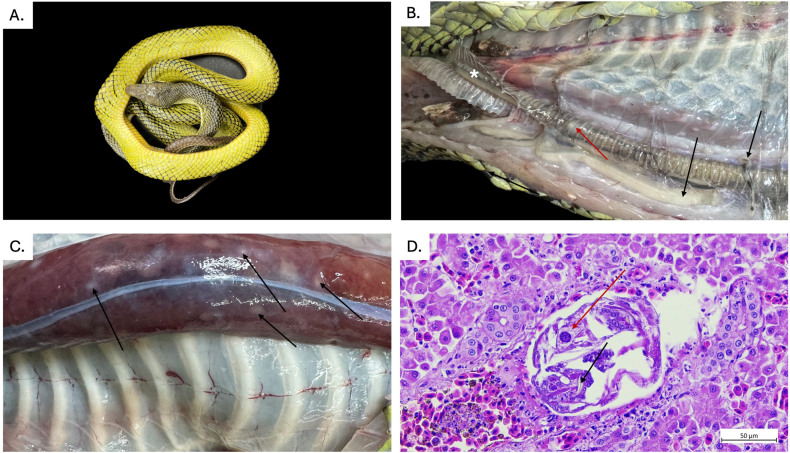
Detection of *Raillietiella orientalis* adults and evidence of organ larval migration in a deceased red-tailed green snake (*Gonyosoma oxycephalum,***A.**). **B.** Adult pentastomids located in the intratracheal (cranial, asterisk) and peritracheal regions, including a large female and a smaller male (black arrows). Macroscopic granulomatous lesions in the trachea are indicated by the red arrow. **C.** Multifocal to coalescing hepatic “milk spot” lesions indicated by black arrows **D.** Tissue lesions in the hepatic parenchyma containing parasitic larvae, morphologically consistent with pentastomids. Notice the sclerotized structure (black arrow) and the eosinophilic gland (red arrow).

**Fig. 5:**
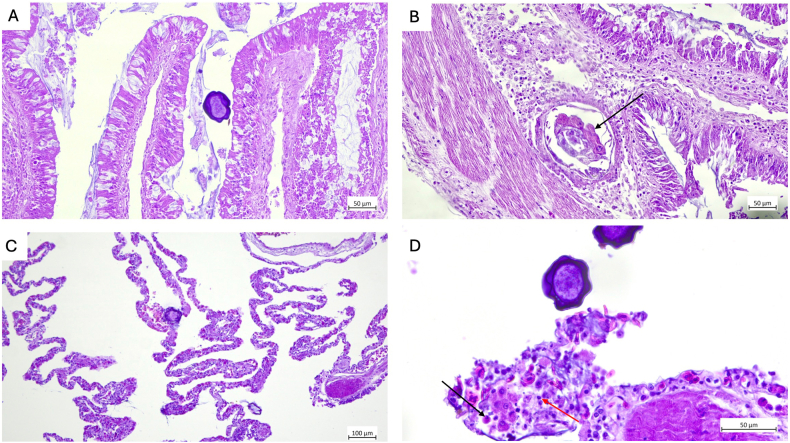
Histological sections showing tissue alterations associated with *Raillietiella orientalis* infection in a red-tailed snake (H&E). **A.** Intestinal mucosa with a pentastomid egg present in the lumen (20x). **B.** Intestinal mucosa showing a pentastomid larva expanding and distorting the mucosal layer (20x). **C.** Lung tissue showing thickening of the faveolar walls; a pentastomid egg is visible within the lumen (10x). **D.** Close-up of faveolar lung tissue exhibiting a lymphocytic (red arrow) – heterophilic (black arrow) infiltrate (40x).

In histological analyses, extensive alterations were observed across multiple organs, being consistent with a high parasitic burden and confirming the findings of numerous adults or migrating larvae as well as pentastomid eggs in the fecal analysis. In the trachea, where adult *R. orientalis* were present, a marked mucosal expansion by a granulomatous infiltrate indicated a chronic, focally destructive host response. The predominance of histiocytes, lymphocytes, and heterophils (syn. granulocytes), together with multifocal mineralization, suggested a rather long-lasting tissue irritation and inflammation most likely caused by attachment, feeding activity, and movement of the parasites. The hepatic parenchyma was multifocally expanded by the presence of pentastomid larvae, resulting in associated lesions that were evident macroscopically as multifocal to coalescing areas (Fig. 4). Intestinal lesions were also pronounced, with mucosal expansion driven by parasitic larvae and associated inflammation. The concurrent presence of nematode larvae likely intensified the mucosal damage, since mixed parasitic infections are known to exacerbate epithelial injury and inflammatory recruitment of heterophils to infection sites. The widespread deposition of pentastomid eggs in the mucosa of all organs suggested active reproduction of adults and significant ectopic egg localization within the host (Fig. 5A and B).

**Fig. 6:**
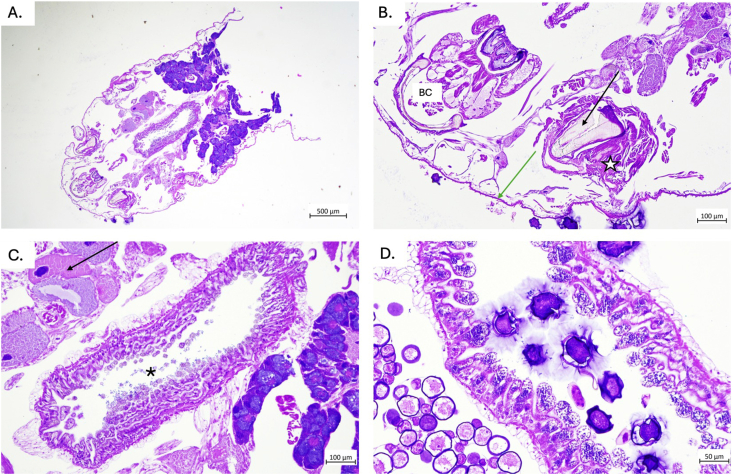
Histologic sections of a female *Raillietiella orientalis,* H&E. **A.** Longitudinal section of a female *R. orientalis*. **B.** Magnification of anterior part showing the buccal cadre (BC), the cuticle (green arrow) as well as the chitinous hooks (black arrow) and the striated muscle around the hooks which control hook retraction (star). **C.** Close-up on the intestinal tract (asterisks) with the eosinophilic glands (black arrow) and the acidophilic glands (red arrow). **D.** Close-up of the oviduct with developing eggs.

Similarly, current pulmonary lesions reflected both direct parasite migration and systemic inflammatory spillover with diffuse interstitial thickening of the faveolar walls by lymphocytic–heterophilic infiltrates. The expansion of the alveolar septa by edema and mixed inflammatory cells likely represented a combination of parasite-induced tissue disruption and secondary inflammatory amplification (Fig. 5C and D). Histological examination of the pentastomids revealed the characteristic internal anatomy of these parasites (Fig. 6).

**Fig. 7:**
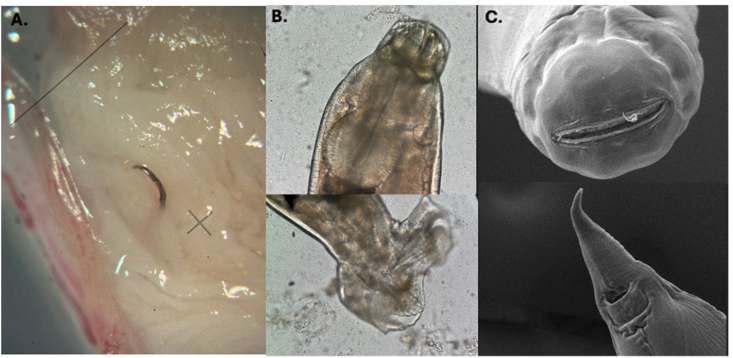
Gross and microscopic views of *Kalicephalus* sp. **A.** Adult Kalicephalic nematode embedded in the intestinal mucosa (2.5x). **B.** Light microscopic image of a male *Kalicephalus* specimen showing the typical arched structure of the buccal capsule and the pronounced bursa copulatrix with spicules (10x). **C.** SEM images of a female *Kalicephalus* sp. displaying the anterior part of the buccal cavity, and the posterior end with visible anal and vulval openings.

Additionally, the nematode *Kalicephalus* sp. was macroscopically observed to be embedded in the intestinal mucosa, thereby showing the distinctive bracket-like stiffeners on the mouth capsule and sexual dimorphism between females and males (Fig. 7). *Post-mortem* fecal examination revealed the presence of *Kalicephalus* sp. eggs in low-grade. Furthermore, bacterial isolation from the liver demonstrated heavy infections by *Proteus vulgaris* and *Pseudomonas aeruginosa*.

## 4 Discussion

Several studies have documented high infection levels and considerable pathogenicity of the pentastomid parasite *R. orientalis* in wildlife reptiles in the United States of America (Miller et al., 2020; Walden et al., 2020; Farrell et al., 2023). However, there are very few reports on captive animals in Europe (Hellebuyck et al., 2025; Sapion-Miranda et al., 2025).

The high parasite burden observed in this case is noteworthy, particularly, as *G*. *oxycephalum* has not previously documented as definitive host for *R. orientalis*, despite both species being indigenous to Southeast Asia (Christoffersen and De Assis, 2013; Dela Cruz et al., 2018). Furthermore, while the host-parasite relationship in the wild remains understudied for this species, the high burden in this case was likely compounded by anthropogenic stressors. Factors such as stress of capture, transit, and subsequent maintenance in captivity may have led to significant immunocompromise (Paré, 2008). Additionally, potential underlying diseases -including co-infections observed in this case-likely contributed to the severity of the infection (Paré, 2008; Walden et al., 2020). The size of the adult pentastomids were similar to the ones described in snakes before (Kelehear et al., 2014; Farrell et al., 2019; Walden et al., 2020; Graham et al., 2026). Notably, the size of female pentastomids differed markedly when compared to other hosts (e.g. lizards) (Hellebuyck et al., 2025; Sapion-Miranda et al., 2025). This discrepancy underscores the remarkable adaptability and phenotypic plasticity of *R. orientalis,* which may facilitate its infections in an extensive host range (e.g. chelonians, lizards, snakes), as suggested elsewhere (Westfall et al., 2019; Walden et al., 2020; Fieldsend et al., 2021; Fonseca, 2021; Hellebuyck et al., 2025; Sapion-Miranda et al., 2025; Palmisano et al., 2026). Furthermore, females, that are substantially larger than their male counterparts, typically exhibit high reproductive outputs, as also evidenced by the large number of eggs observed in current histological analyses. The high reproductive output of *R. orientalis* may also form part of its epidemiological success by spreading into previously non-endemic geographic areas (Miller et al., 2020; Walden et al., 2020; Sapion-Miranda et al., 2025).

Overall, the histopathological findings align well with multi-organ *R. orientalis* infections from literature (Kelehear et al., 2014; Graham et al., 2026; Palmisano et al., 2026) and illustrate that high parasite loads cause a combination of mechanical damage, granulomatous inflammation, and larval migration across organ systems. The observation of an adult female pentastomid leaving the snake through the oral cavity, as reported by the snake's owner, has previously been described by Self and Kuntz (1967), who noted that captive snakes infected with pentastomids frequently coughed-up or regurgitated adult parasites. This behavior may be considered as a kind of stress response, since these parasites tend to abandon stressed and/or hypoxic hosts (Self and Kuntz, 1967; Self, 1972). During necropsy, several specimens were observed obstructing the trachea and extending towards the oral cavity, further supporting these earlier observations in agreement with other case reports (Kelehear et al., 2014; Walden et al., 2020). While this finding indicates a potential for mechanical airway obstruction, it remains unclear whether parasite accumulation occurred *ante mortem* and resulted in asphyxiation, or whether migration into the trachea occurred *post-mortem*. However, reduced pulmonary functions due to obstructions have been documented for pentastomiasis (Riley, 1986; Miller et al., 2018), and related histologic findings were comparable with pneumonia-related alterations described in other reptilian hosts infected with pentastomids and other parasites (Jacobson, 2020; Walden et al., 2020; Plumeriastuti et al., 2023). The presence of pentastomid larvae within the hepatic parenchyma demonstrated the systemic nature of the infection, as these intralesional larvae reflect somatic migration that forms part of the life cycle of pentastomids, ultimately resulting in mature adults (Riley, 1986; Miller et al., 2017). This is corroborated by the detection of pentastomid eggs not only in the intestinal mucosa but also within the liver, lungs, and tracheal mucosa, highlighting widespread dissemination. The isolation of bacteria from the liver further suggests that parasite-associated tissue damage may have predisposed to secondary bacterial infection.

Interestingly, despite centrifugation with Sheather's sugar solution, egg recovery remained markedly low during flotation when compared with DSFM. This observation agrees with Palmisano (2025), who reported Sheather's solution as significantly less effective than other flotation media, such as sodium nitrate and zinc sulfate, and found that wet mounts were superior to flotation techniques by resulting in a 100% egg detection for *R. orientalis* diagnosis (Palmisano et al., 2025b). This discrepancy is likely attributable to the physical characteristics of pentastomid eggs, which are relatively dense, thick-shelled, and often embryonated, potentially limiting their flotation efficiency. Pentastomid eggs have previously been detected using both sedimentation and flotation techniques (Wolf et al., 2014), however, the low number of positive cases in the former study precluded a statistical evaluation of diagnostic method sensitivities (Palmisano et al., 2025b). In contrast, pentastomid eggs were shown to exhibit positive buoyancy by Palmisano et al. (2025b), which may argue against the routine use of sedimentation techniques alone. However, these diagnostic evidences should principally be considered when analyzing fecal samples from reptiles with suspicion of pentastomiasis and underscore the importance of performing a native (direct) fecal smear as an initial diagnostic step, given its simplicity and high sensitivity (Palmisano et al., 2025b).

In the current case*, Kalicephalus* sp. infection was initially considered an incidental finding, as this nematode is commonly reported in squamates and infections often remain subclinical (Schad, 1962; Frank, 1986; Hallinger et al., 2020). However, its clinical relevance is likely dependent on the infection intensity; while low burdens may be tolerated, heavy parasitic burdens are known to cause severe hemorrhagic and necrotizing enteritis (Frank, 1986; Hallinger et al., 2020). The moderate-grade infection observed here, characterized by macroscopic lesions in the internal mucosa (Fig. 7), likely represented a significant metabolic and immunological drain to the host. Gastrointestinal helminths have been shown to modulate the hosts immune environment, often enhancing susceptibility to secondary parasitic infections (Kloosterman et al., 1989; Bickle et al., 2008; Hananeh et al., 2022). While specific studies on the immune responses of reptiles during helminth infections are currently lacking, the high pentastomid burden observed suggests a synergistic pathological relationship. These findings imply that *Kalicephalus* sp. should not be dismissed as a background finding; rather its presence may indicate an altered immune response that facilitated the establishment of a high systemic parasitic burden. This suggests that co-infections should be evaluated as interacting disease processes rather than as isolated conditions. For example, in North America, *R. orientalis* is often accompanied by ophidiomycosis (snake fungal disease) and/or mixed parasitism, illustrating how multiple pathogens can converge to compromise host health (Walden et al., 2020; Graham et al., 2026)

Anthropogenic translocation via the pet trade represents a significant mechanism for pathogen introduction into novel ecosystems (Travis et al., 2011; Rush et al., 2021; Murray et al., 2025). The current case report relied on parasite infections in a wild-caught and imported animal. This case mirrors recent developments in North America, where infected snakes in the pet trade have been transported to unsuspecting buyers (Farrell et al., 2023; Palmisano et al., 2026). A major problem within the current international exotic pet trade practice is that a substantial proportion of organizations operate illegally, and even when trade is legal, veterinary surveillance in exotic medicine is extremely limited (Kerwick et al., 2008; Mitchell, 2011; Nijman, 2021). Under these conditions, invasive parasites as well as other pathogens, such as fungi, bacteria and viruses may spill over, causing problems not only within the hobby herpetoculture but also in conservation settings, which often rely on strict isolation protocols to protect already vulnerable populations from emerging infectious diseases (Jacobson, 1993; Rush et al., 2021).

When exotic reptiles are intentionally released or accidently escape into the wild, they may act as reservoir hosts for parasites not previously present in the region (Corn et al., 2014). A critical factor in the transmission and spread of *R. orientalis* is the involvement of cockroaches (Blattodea) as intermediate hosts (Bosch, 1986; Palmisano et al., 2022; Sapion-Miranda et al., 2025). The coprophagous nature of many synanthropic cockroaches suggests a broad range of potential suitable intermediate hosts (Gałęcki and Sokół, 2019; Patel et al., 2022). Furthermore, the risk may be amplified if infection can occur without the involvement of a secondary intermediate or paratenic host, as the direct ingestion of infected cockroaches could represent a viable biological bridge between imported reptiles and European ecosystems. Such a pathway may help explain the occurrence of *R. orientalis* in captive-kept insectivorous reptiles (Hellebuyck et al., 2025; Palmisano et al., 2026) and may indicate greater flexibility in the parasites transmission ecology than currently assumed, particularly following its recent detection in an Aldabra giant tortoise (Palmisano et al., 2026).

## 5 Conclusion

This report highlights yet another case of *R. orientalis* introduction to Europe through the global wildlife trade. While the detection of this infection in an imported *G. oxycephalum* from Indonesia raises valid concerns for herpetoculturists and zoological institutions, the risk of spillover into the European wild fauna remains speculative, particularly given the limited understanding of the parasites life cycle flexibility and transmission ecology. Nevertheless, the broad host spectrum of this euryxenous parasite suggest a potential pathway for infection if contact with native fauna occurs.

Crucially, this case report underscores that the clinical outcome of such introductions may be exacerbated by co-exciting pathogens. Until further data on wild populations and reptilian immune responses are available, implementing stricter quarantine protocols and routine parasitological screenings remains a precautionary key measure. Further research into reptile eco-immunology is essential to better understand how co-infections influence the success and pathogenicity of emerging neozoans, ultimately helping to limit their introduction and impact on native biodiversity.

## Funding

This research did not receive any specific grant from funding agencies of public-, commercial-, or not-for-profit-sectors.

## CRediT authorship contribution statement

**Paula Sapion-Miranda:** Conceptualization, Formal analysis, Investigation, Visualization, Writing – original draft. **Ulrich Gärtner:** Formal analysis, Resources, Visualization. **Anja Taubert:** Supervision, Writing – review & editing. **Carlos Hermosilla:** Resources, Supervision, Writing – review & editing. **Fanny Kratz:** Formal analysis, Investigation, Writing – original draft. **Malek J. Hallinger:** Supervision, Writing – review & editing.

## Conflict of interest

The authors declare no conflict of interest.
